# Correlation between fungal sIgE and bronchial asthma severity

**DOI:** 10.3892/etm.2013.1153

**Published:** 2013-06-10

**Authors:** HUI ZOU, LEI SU, QIU-HONG FANG, YING-MIN MA

**Affiliations:** Department of Pulmonary and Intensive Care Unit, Beijing Shijitan Hospital, Capital Medical University, Beijing 100038, P.R. China

**Keywords:** asthma, fungal sensitisation, specific immunoglobulin E

## Abstract

Fungal sensitisation is closely associated with asthma; however, the correlation between fungi and asthma severity remains unclear. The aim of this study was to investigate the severity of asthma in 100 patients with asthma due to fungal and non-fungal allergens. A total of 100 outpatients and inpatients with asthma were selected from 2010 to 2011 and were divided into three groups (mild, moderate and severe) according to their clinical manifestations, lung function results and treatment situations. Enzyme-linked immunosorbent assay (ELISA) was used to detect the levels of specific immunoglobulin E (sIgE) to five fungal allergens and seven non-fungal allergens in the serum of all patients. The levels of sIgE to *Aspergillus*, *Penicillium* and *Candida albicans* allergens in the severe group were significantly higher compared with those in the moderate and mild groups (P<0.001 and P<0.05, respectively); those of the moderate group were significantly higher compared with those of the mild group (P<0.05). No significant difference was observed for the levels of sIgE to *Alternaria alternata* among the three groups. sIgE to *Cladosporium herbarum* was not present for all three groups. No significant difference was observed for the levels of sIgE to non-fungal allergens among the three groups. Fungal allergens are closely correlated with the severity of asthma, whereas non-fungal allergens are not.

## Introduction

Severe or intractable asthma is a common clinical problem and exposure to particular allergens and sensitisation to these allergens have an important role in its pathogenesis (1). Fungi are closely associated with asthma and studies suggest that the mortality, hospitalisation rate, respiratory symptoms and peak expiratory flow (PEF) of patients with asthma are associated with a high fungal spore content in outdoor air (2–6). Fungi, including *Alternaria alternata*, *Penicillium*, *Aspergillus* and *Cladosporium*, are common allergens of patients with asthma (7). Sensitisation to these fungal allergens is related to the acute aggravation and severity of asthma (8–11). Among patients with life-threatening asthma, patients who are positive in the dermal sensitivity test to *A. alternata* are extremely common (12).

Few studies concerning the correlation of fungal sensitisation with asthma severity exist and the correlation remains unclear. Moreover, the distribution of allergenic fungi varies in different regions. Considering the close association of fungal allergens with asthma, we selected cases of outpatients and inpatients with asthma in Beijing Shijitan Hospital from 2010 to 2011 to detect the levels of specific immunoglobulin E (sIgE) to various fungal and non-fungal allergens and investigate the correlation of fungal sensitisation with asthma severity in the local region. This study is likely to be significant in the prevention and treatment of severe asthma.

## Subjects and methods

### Case collection

A total of 100 cases of outpatients and inpatients diagnosed with bronchial asthma were selected from 2010 to 2011. All patients complied with the diagnostic criteria in the Prevention and Treatment Guideline of Bronchial Asthma prepared by the Asthma Study Group under the Respiratory Disease Branch of the Chinese Medical Association in 2008. According to the clinical manifestations of the patients, lung function prior to treatment and treatment modes for maintaining asthma control, the asthma severity of the patients was classified into mild severity (grades 1 and 2), moderate severity (grade 3) and severe severity (grade 4) (13). Patients with chronic obstructive pulmonary disease, complicated active, acute or chronic lung diseases, severe autoimmune diseases, therioma, coronary heart disease and hypertension were excluded. Pregnant and lactating women were also excluded. Horizontal comparisons of age, gender, disease history, lung function, total IgE (tIgE) and sIgE were conducted for all patients. This study was conducted in accordance with the Declaration of Helsinki and with approval from the Ethics Committee of Beijing Shijitan Hospital. Written informed consent was obtained from all participants.

### sIgE detection

A refrigerated sIgE kit (FOOKE GmbH, Borken, Germany) was used and maintained at room temperature for 30 min. The concentrated washing solution (20 times) was diluted with distilled water for repeated use. An adequate number of enzyme-encapsulated plates was fixed onto the frame. The standard, test sample and blank control wells were set up and the positions of the various wells were recorded. In the standard well, 50 *μ*l standard solution was added. In the test sample well, 10 *μ*l test sample solution was initially added (five times the test sample dilution solution). No solution was added to the blank control well. Then, the plate was incubated in an incubator at 37°C for 30 min. The liquid in each well was removed and the plate was dried with bibulous paper. Subsequently, each well was filled with washing solution. After standing for 1 min, the washing solution was removed, the plate was dried with bibulous paper and the plate was washed repeatedly four times. Approximately 50 *μ*l enzyme working fluid was added to each well, with the exception of the blank control well. The plate was incubated at 37°C for 30 min. The liquid in each well was then removed and the plate was dried with bibulous paper. Subsequently, each well was filled with washing solution. After standing for 1 min, the washing solution was removed, the plate was dried with bibulous paper and the plate was washed repeatedly four times. Approximately 50 *μ*l chromogenic agent liquid A was placed into each well and then 50 *μ*l chromogenic agent liquid B was added. After the plate had been agitated on the flat mixing device for 30 sec, development was conducted at 37°C for 15 min in the dark. Then, the enzyme-linked immunosorbent assay (ELISA) plate was removed and 50 *μ*l termination solution was added to each well to terminate the reaction. Zone adjustment was conducted in the blank well. The absorbance values [optical density (OD)] of the various wells were measured at 450 nm within 15 min of the termination of the reaction. The linear regression equation of the standard curve was calculated according to the standard concentrations and corresponding OD values. The corresponding sample concentration was calculated based on the sample OD value using the linear regression equation. The final concentration is calculated as the product of the measured concentration multiplied by the dilution factor.

### Statistical analysis

SPSS 17.0 (SPSS, Inc., Chicago, IL, USA) was used for statistical analysis. Measurement data are expressed as mean ± standard deviation. One-factor analysis of variance was used for comparison of means among multiple groups, whereas the Chi-square test was used for comparison of classification data. P<0.05 was considered to indicate a statistically significant difference.

## Results

### Clinical features of the patients

For the clinical features of the three groups of patients with asthma, patients in the mild group and the moderate group were significantly younger compared with those in the severe group (P=0.015, P=0.0001). No significant differences among the various groups were observed in terms of gender, asthma onset age < 40 years old, smoking, allergic rhinitis disease history and positive family history of asthma. In the clinical examinations, no significant difference was noted for tIgE, eosinophil percentage, forced expiratory volume in 1 sec (FEV1) and FEV1/forced vital capacity (FVC) among the three groups (Table I).

### Allergen distributions

The percentage of patients with asthma sensitised by fungal allergens, including *Penicillium*, *Aspergillus* and *C. albicans,* was significantly higher in the severe group than in the moderate and mild groups; the percentage in the moderate group was significantly higher compared with that in the mild group (P<0.05). For *A. alternata*-sensitised patients, no significant difference was observed in the percentages among the three groups. Patients with asthma caused by *C. herbarum* were not observed in the three groups. The percentage of patients sensitised by any of the five fungi was significantly higher in the severe group than in the moderate and mild groups; the percentage in the moderate group was significantly higher compared with that in the mild group (P<0.001). In the severe group, almost half of the patients with asthma were sensitised by any of the fungi. For patients simultaneously sensitised by multiple fungi, no significant difference was observed in the percentages among the three groups.

For patients sensitised by non-fungal allergens, including *Dermatophagoides pteronyssinus*, house dust, cat hair, dog hair, *Artemisia argyi* and a tree combination (maple/birch/beech/oak/Chinese parasol/poplar), no significant difference was noted in the percentages among the three groups. For patients sensitised by any of the seven non-fungal allergens, no significant difference existed in the percentages among the three groups. Moreover, no significant difference was observed in the percentage of patients simultaneously sensitised by multiple non-fungal allergens among the three groups. The percentage of patients sensitised by combined fungal and non-fungal allergens was significantly higher in the severe group than in the moderate and the mild groups; the percentage in the moderate group was significantly higher compared with that in the mild group (P<0.001; Table II).

### ELISA

**Figure 1.**
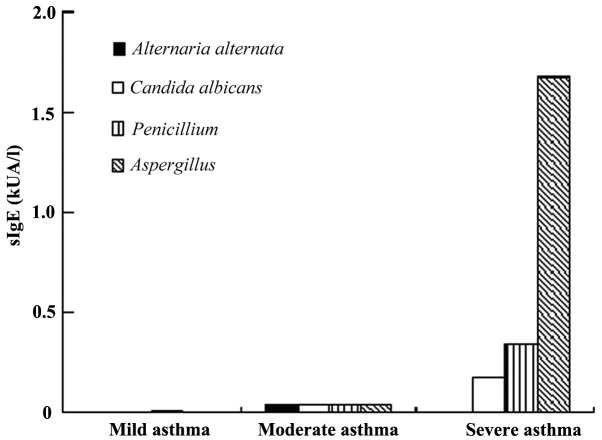
sIgE of fungi sensitinogen in the three asthma groups. sIgE, specific immunoglobulin E.

The levels of sIgE to *Aspergillus*, *Penicillium* and *C. albicans* of the patients with asthma in the severe group were significantly higher than those in the moderate and mild groups; those of the moderate group were significantly higher than those of the mild group (P<0.01 or P<0.05). Regarding the level of sIgE to *A. alternata*, no significant difference was observed among the three groups of patients with asthma. sIgE to *C. herbarum* among the three groups was not present. Regarding the levels of sIgE to seven non-fungal allergens, no significant difference was noted among the three groups of patients with asthma (Table III, Fig. 1).

## Discussion

The results of the current study demonstrated that fungal sensitisation is common in patients with severe asthma and fungal allergens, including *Aspergillus*, *Penicillium*, and *C. albicans*, in the local region are evidently related to the severity of asthma in the patients. The results of the study are in line with the results of previous studies on the correlation of fungi with asthma severity. A 30-center survey on community respiratory health in Europe demonstrated that asthma severity increases with the increase of sensitisation frequency caused by *A. alternata* or *C. herbarum* (11). The survey also indicated that the percentage of patients sensitised by one or more allergens, including *A. alternata*, *C. herbarum*, *Epicoccum* and *Helminthosporium*, in a group of patients treated for acute aggravation of asthma in an intensive-care unit (ICU) reached 54%. However, the percentage of patients sensitised by fungi in the other group of patients with asthma who were not admitted in the ICU was only 30% (8). An additional study demonstrated that *A. alternata* sensitisation is a risk factor for the development of severe asthma (10).

The results of the current study indicate that common *A. alternata* sensitisation is not correlated with asthma severity, in opposition to the results of previous reports. Asthma patients sensitised by *C. herbarum* were not observed in the three groups. Given the different geographical environments, climates and vegetations, greater differences exist in the distribution of allergenic fungi between regions (14). A study reported that *Penicillium* and *Paecilomyces variotii* were the dominant colonies of fungi distributed in the air in Beijing, which was in line with the distribution of fungi in the environment, wherein *Aspergillus* yeast and *A. alternata* were the dominant colonies. In the literature, *A. alternata* is not reported to be the dominant fungus in the air in Beijing. In the literature, *A. alternata* is not reported to be the dominant fungus in the air in Beijing. Moreover, the data demonstrated that the distribution of *C. herbarum* is not detected in Beijing (15). These findings may be attributed to the fact that A. alternata is not a dominant fungus in this area. Therefore, fewer patients with asthma are allergic to *A. alternata*. Given that *C. herbarum* content is undetectable in the air, the possibility of *C. herbarum* sensitisation is extremely minimal.

The results of studies concerning the correlation of non-fungal allergens with disease severity in patients with asthma are inconsistent. A number of studies suggested that certain non-fungal allergens, including dog hair, are associated with asthma severity, whereas other studies suggested that no correlation existed between these components (9,16). In the present study, the levels of sIgE to seven common non-fungal allergens were detected and the results show that the percentage of patients sensitised by non-fungal allergens (8–42%) is higher than that of patients sensitised by fungal allergens (4–25%). The levels of sIgE to non-fungal allergens are evidently higher compared with those to fungal allergens. The sensitisation to non-fungal allergens is stronger than that to fungal allergens and the sensitisation to non-fungal allergens is common in all patients with asthma. However, non-fungal allergens are not related to the disease severity of patients with asthma.

The difference between fungal and non-fungal allergens lies in the complex fungal allergen components. Fungal allergens include proteases, glycosidases, protein product components, oxidative stress-related proteins and enzymes involved in glyconeogenesis or the pentose phosphate pathway. Proteases and glycosidases directly affect the host, whereas the other three components function in the metabolism process of spore germination. Therefore, the exposure to fungal spores is equivalent to the exposure to all fungal allergens and not just the exposure to simple allergens, including non-fungal allergen pollen protein or animal pelage. In addition, fungi are common in the environment; therefore, the human respiratory tract is often exposed to fungal spores in the air. The most common airborne fungi include *C. herbarum*, *A. alternata* and *Aspergillus*. Fungal and non-fungal allergens, including *D. pteronyssinus*, dog hair and pollen, are protein allergens. However, fungal allergens have unique features; they constantly germinate and infect the host or implant in the respiratory tract of the host. Therefore, fungi generate non-allergenic toxins and enzymes to pathogenic bacteria by stimulating the defense system of the host. These substances have an auxiliary role in sensitisation stimulation and cause a greater impact on the host.

Fungi, including *C. albicans* and *Tracheophyta*, are common colonisation fungi of the skin or gastrointestinal tract. Although *C. albicans* is not an airborne fungus, reports have indicated that ∼10% of patients with mild asthma and almost 33% of patients with severe asthma are sensitised by this fungus (9). Trichophyton is not an airborne fungus; however, it often causes skin and fingernail infections. One study indicated that after the body absorbs fungal antigens, the body generates an IgE antibody to induce air tract sensitisation and thus, cause asthma occurrence (17). Therefore, non-airborne fungi also induce sensitisation in the body, thereby causing asthma occurrence. The present study also demonstrated that among patients with asthma, certain moderate and severe asthma patients are sensitised by *C. albicans*, which is significantly correlated with asthma severity.

The effect of antifungal therapy on severe asthma remains controversial and relevant studies are rare. In a retrospective study, the hospitalisation rate and glucocorticoid treatment course of *Aspergillus*-allergenic patients with severe asthma who failed to comply with the diagnostic criteria of allergenic bronchopulmonary *Aspergillus* was evidently reduced following treatment with itraconazole for several months. However, no change was observed in the tIgE and sIgE levels (18). In another study, fluconazole (100 mg/day) was administered for five months (once a day) to *Tracheophyta*-sensitised patients with severe asthma. Fluconazole reduced the bronchial hyper-reactivity of patients to inspiratory *Tracheophyta*, reduced the hormone requirement and increased the PEF value (19). Moreover, another study used itraconazole to treat fungus-sensitised patients with severe asthma and identified that itraconazole appropriately enhanced the quality of life of the patients, improved the rhinitis score, increased the PEF value and reduced the tIgE level (20). These studies suggest that antifungal therapy partially helps fungus-sensitised patients with severe asthma; however, large-scale clinical trials are required for confirmation.

The limitation of this study is the small number of patients with asthma; thus, confirmation from large-scale clinical trial data is required.

This study demonstrated that fungal sensitisation is closely correlated with disease severity in patients with asthma by detecting the levels to sIgE to different fungi in patients. Non-fungal allergens are not correlated with the disease severity of patients with asthma. Moreover, this study demonstrated that fungi have an important role in the disease severity of patients with asthma. Therefore, this study is significant for the future prevention and treatment of severe asthma.

## Figures and Tables

**Table I.** Clinical features of the three groups of patients with asthma.

Characteristics	Mild asthma	Moderate asthma	Severe asthma	P-value
Cases (n)	52	24	24	
Age (years)	50±15.67	54±11.80	68.75±12.51^a,b^	<0.001^a^, <0.05^b^
Gender (% female)	65	58	75	>0.05
Asthma onset age <40 years (%)	58	42	75	>0.05
Smoking (%)	19	8	33	>0.05
Allergic rhinitis (%)	54	42	33	>0.05
Family history (%)	46	33	25	>0.05
tIgE (U/ml)	281.63±594.18	427.98±669.62	582.02±981.31	>0.05
Eosinophil (%)	4.51±4.83	7.29±6.89	4.90±5.77	>0.05
FEV1 (litres)	60.75±24.62	63.00±17.13	46.33±7.34	>0.05
FEV1/FVC (%)	63.89±15.98	61.05±14.53	52.67±8.38	>0.05

Data for age, tIgE, eosinophils, FEV1 and FEV1/FVC are expressed as mean ± standard deviation.

a Mild group vs. severe group;

b moderate group vs. severe group. tIgE, total immunoglobulin E; FEV1, forced expiratory volume in 1 sec; FVC, forced vital capacity.

**Table II.** Distributions of fungal and non-fungal allergens among the three groups of patients with asthma (%).

	Mild asthma (n=52)	Moderate asthma (n=24)	Severe asthma (n=24)	P-value
Fungal allergens				
*Aspergillus*	<0.01	8	25	<0.05
*Penicillium*	4	8	25	<0.05
*Alternaria alternata*	<0.01	8	<0.01	
*Cladosporium herbarum*	0	0	0	
*Candida albicans*	<0.01	8	25	<0.05
Any one fungal sensitisation	4	8	50	<0.001
>1 fungal sensitisation	<0.01	8	17	
Non-fungal allergens				
*Dermatophagoides pteronyssinus*	12	33	17	
*Dermatophagoides culinae*	15	17	17	
House dust	23	42	33	
Cat epithelium	12	33	8	
Dog epithelium	15	42	8	
*Artemisia argyi*	12	17	17	
Tree combination	<0.01	8	<0.01	
Any one non-fungal sensitisation	35	67	33	
>1 non-fungal sensitisation	8	25	8	
Combined fungal and non-fungal sensitisation	<0.01	8	50	<0.0001

Tree combination, maple/birch/beech/oak/Chinese parasol/poplar.

**Table III.** Levels of sIgE to fungal and non-fungal allergens of the three groups of patients with asthma.

	sIgE(kUA/l)			P-value
	Mild asthma (n=52)	Moderate asthma (n=24)	Severe asthma (n=24)	
Fungal allergens				
*Aspergillus*	<0.01±0.00	0.04±0.13	1.68±3.40^a,b^	<0.01^a^, <0.05^b^
*Penicillium*	0.01±0.07	0.04±0.15	0.34±0.84^a,b^	<0.05^a^, <0.05^b^
*Alternaria alternata*	<0.01±0.00	0.04±0.13	<0.01±0.00	
*Cladosporium herbarum*	0	0	0	
*Candida albicans*	<0.01±0.00	0.04±0.12	0.17±0.33^a,b^	<0.01^a^, <0.05^b^
Non-fungal allergens				
*Dermatophagoides pteronyssinus*	3.60±16.52	0.23±0.38	0.25±0.76	
*Dermatophagoides culinae*	0.99±4.11	0.14±0.33	0.37±1.12	
House dust	3.95±12.36	1.03±2.13	1.51±2.98	
Cat epithelium	2.33±9.93	0.30±0.49	0.10±0.34	
Dog epithelium	1.20±3.85	2.53±5.73	1.78±6.19	
*Artemisia argyi*	1.07±3.68	0.14±0.34	0.74±2.32	
Tree combination	<0.01±0.00	0.05±0.18	<0.01±0.00	

Data are presented as mean ± standard deviation.

a Mild vs. severe group;

b moderate vs. severe group. sIgE, specific immunoglobulin E.
